# FSC231 alleviates paclitaxel‐induced neuralgia by inhibiting the interactions between PICK1 and GluA2 and activates GSK‐3β and ERK1/2

**DOI:** 10.1002/brb3.2380

**Published:** 2021-09-28

**Authors:** Xi Zhang, Jiagao Wang, Ran Ran, Yuchuan Peng, Yun Xiao

**Affiliations:** ^1^ Department of Anesthesiology Renmin Hospital Hubei University of Medicine Shiyan P. R. China

**Keywords:** FSC231, GluA2, GSK‐3β, paclitaxel, PICK1

## Abstract

**Background:**

FSC231, a PSD‐95/DLG/ZO‐1 (PDZ) domain inhibitor of protein kinase Cα interacting protein 1 (PICK1), has analgesic effects, but the mechanism remains unclear.

**Methods:**

The expression level of PICK1 in dorsal root ganglion (DRG) of rats was changed by vector plasmid, and the effect of PICK1 on paclitaxel (PTL)‐induced neuralgia of rats was observed in collaboration with FSC231 treatment. The possible molecular mechanisms were explored by quantitative real‐time polymerase chain reaction (qRT‐PCR), Western Blot and co‐immunoprecipitation (Co‐IP) techniques.

**Results:**

PTL treatment can significantly reduce mechanical withdrawal threshold (MWT), shorten thermal withdrawal latency (TWL), promote DRG inflammation and release of substance P (SP), stimulate PICK1 expression, decrease α‐amino‐3‐hydroxy‐5‐methyl‐4‐isoxazole‐propionic acid receptor 2 (AMPAR, GluA2) level and increase glycogen synthase kinase‐3β (GSK‐3β) and extracellular regulated protein kinases1/2 (ERK1/2) phosphorylation in rats, while FSC231 treatment can alleviate the above effects induced by PTL. Overexpression of PICK1 can counteract reduced PICK1 level, increased GluA2 level and decreased GSK‐3β and ERK1/2 phosphorylation levels caused by FSC231 treatment. The results of Co‐IP confirmed the interactions between PICK1 and GluA2. Both FSC231 treatment and silent PICK1 improved PTL‐induced MWT reduction, TWL shortening, inflammation, SP release and related gene expression changes, with cumulative effect.

**Conclusion:**

FSC231 activates GSK‐3β/ERK1/2 by inhibiting the interaction between PICK1 and GluA2 and alleviates PTL‐induced DRG neuralgia in rats.

## 1 INTRODUCTION

Paclitaxel (PTL) is a member of taxanes. As a first‐line anti‐tumor drug in clinical practice, it has significant therapeutic effects on various malignant tumors and has been widely used in cancer patients. However, dose‐dependent neurotoxicity is present during the course of treatment, most commonly chemotherapy‐induced neuropathic pain (CIPN) (Xiao & Bennett, 2012). CIPN is characterized by numbness, tingling or flashing pain in the distal extremities. It is concentration dependent and may persist for months or even years after treatment. At present, the mechanism of PTL neuropathic pain has not been clearly reported, and it is not sensitive to the current analgesic drugs, and there is a lack of effective treatment means, which further affects the effect of tumor treatment and the quality of life of patients (Stage et al., 2018), restricting the clinical application of PTL. Therefore, it is of profound clinical significance to explore the pathogenesis of PTL‐induced neuralgia and to find effective treatment methods. Therefore, putting forward effective prevention and treatment methods for this kind of neuralgia is of great significance to improve the quality of life and survival rate of tumor patients, and it is an important subject to be solved urgently at present.

Protein interacting with Cα kinase 1 (PICK1) is widely distributed in human and animal tissue cells. The PSD‐95/DLG/ZO‐1 (PDZ) domain and the Bin/Amphiphysin/RVS (BAR) domain of PICK1 are capable of interacting with multiple proteins to form a protein recruitment structure that recruits multiple proteins to specific sites and thus participate in the regulation of various cellular functions and signal transductions, such as pain, schizophrenia, Parkinson's disease, cancer and other diseases occur, and play an important role in the above diseases (Morita et al., 2004). PICK1 expression is associated with pain in dorsal root ganglion (DRG) and spinal posterior horn neurons, and PICK1 is also associated with peripheral nerve injury‐induced neuropathic pain, and it could be a potential biochemical target for the treatment of this disease (W. Wang et al., 2011). In the PDZ region of PICK1, a small molecule inhibitor FSC231 showed significant analgesic effects on chemically stimulating pain, inflammatory pain and neurogenic pain, suggesting that PICK1 could be a new target for analgesia, and the PICK1 inhibitor FSC231 could be a candidate for analgesia (G. Wang et al., 2021). Previous studies of our team confirmed that FSC231 can reduce the expression of PICK1 of DRG in PTL‐induced neuralgia rats and alleviate neuralgia. This study aims to explore the molecular mechanism by which FSC231 inhibits PTL‐induced neuralgia, so as to provide new targets and ideas for the clinical development of effective analgesic drugs and the exploration of intervention measures, and to provide a more sufficient experimental basis for the clinical application of PTL in the treatment of malignant tumors.

## 2 MATERIALS AND METHODS

### 2.1 Animals and groups

Wild‐type male C57BL/6 rats weighted 23–26 g were obtained from Experimental Animal Center of Peking University. Rats were housed under the following conditions: temperature, 21°C; humidity, 40–60%; 12 h light/dark cycle and free access to food and water. Forty C57BL/6 rats aged 8–10 weeks were randomly divided into eight groups (*n* = 10): the control group (CTRL, intraperitoneal injection of normal saline), the FSC231 group (FSC, intraperitoneal injection of FSC231), the paclitaxel group (PTL, intraperitoneal injection of paclitaxel), the FSC231 add paclitaxel group (FSC+PTL, intraperitoneal injection of FSC231 and paclitaxel), the paclitaxel add PICK1 negative control transfection group (PTL+Ad‐shNC, intraperitoneal injection of paclitaxel and PICK1 negative control vector), the paclitaxel add PICK1 silencing vector transfection group (PTL+Ad‐shPICK1, intraperitoneal injection of paclitaxel and PICK1 silencing vector), the FSC231 add paclitaxel and PICK1 negative control transfection group (FSC+shNC+PTL, intraperitoneal injection of FSC231, paclitaxel and PICK1 negative control vector) and the FSC231 add paclitaxel and PICK1 silencing vector transfection group (FSC+shPICK1+PTL, intraperitoneal injection of FSC231, paclitaxel and PICK1 silencing vector). All experimental procedures were carried out in accordance with National Institutes of Health guidelines for the care and use of Laboratory animals (NIH Publications No. 8023, revised 1978) and approved by the Animal Ethics Committee of our hospital.

### 2.2 Rats model establishment

PTL (Sinopharm Group Co. Ltd., China) was dissolved in 1:1 anhydrous alcohol and hydrogenated castor oil, and then diluted with 0.9% normal saline to 0.2 mg/ml before each administration as PTL intraperitoneal injection. The drug was administered at the same time point on day 1, 3, 5 and 7 in the experiment for a total of four times with a total dose of 8 mg/kg.

FSC231 (Merck KGaA, Germany) was dissolved in dimethyl sulfoxide (DMSO) and prepared into a 15.70 μg/ml solution by adding normal saline. FSC231 was intraperitoneally injected at the same time point every day from the first to seventh day of the experiment, 78.40 μg/kg seven times in total. Intraperitoneal injection of FSC231 was completed 3 h before the intraperitoneal injection of PTL.

### 2.3 Virus administration

PICK1 shRNA scramble or PICK1 shRNA (1 nmol/10 μl, Ribobio, Guangzhou, China) was intrathecally injected into L4‐L6 subarachnoid space of the spinal cord of rats to knockdown the PICK1 gene 30 min before the application of PTL.

### 2.4 Detection of paw withdrawal mechanical threshold

Paw withdrawal mechanical threshold was measured before and after modeling for a total of 5 weeks. Von Frey filament (1.0 × 10^15^ g) was used to give a vertical stimulus to the center of the hind leg for 5–10 s. Positive response was defined as the observation of apparent paw withdrawal reflex and paw licking behavior.

### 2.5 Detection of paw withdrawal thermal latency

A white surgical tray was placed in a 55°C thermostat water bath, where a thermometer was inserted to measure the temperature. A transparent glass cover with the top open was placed over it. Rats became gradually quiet after adapting to the new environment about 30 min later. Then, rats were placed in the transparent glass cover and timing began with a stopwatch. Paw withdrawal thermal latency was defined as the time from hind leg contacting with the tray to tiptoeing, withdrawal, paw licking and struggling. To avoid tissue injury, the experiment was stopped if no reflex was elicited within 40 s. Three measurements were performed for each rat with an interval of 5 min, and the average was taken.

### 2.6 Preparation of dorsal root ganglions

After the behavioral measurement, rats were anesthetized by intraperitoneal injection of 1% pentobarbital sodium 40 mg/kg. The rat was fixed in the supine position, the chest was cut open to expose the heart, the perfusion needle was inserted through the apex to the left ventricle and the right auricle was cut open to form the perfusion drainage channel. First, rats were perfused with normal saline at 37°C rapidly. At the same time, rats’ limbs, liver and intestine were observed until the limbs turned white, the liver became pale and the intestine became swollen. The perfusion liquid was changed to 4% paraformaldehyde for tissue fixation. When rats’ limbs were suddenly strained and tail curls were observed, the irrigation velocity was reduced, and the total dosage of 4% paraformaldehyde was about 400 ml. After the completion of perfusion fixation, DRG at the enlarged lumbar segment of the spinal cord was rapidly extracted and soaked in 4% paraformaldehyde for preservation.

### 2.7 Primary neuronal cell culture

Rats were sacrificed on day 14. L4−6 DRGs were harvested and dissociated using collagenase type 1 and dispase (Gibco, USA). DRG neurons were cultured in Dulbecco's modified eagle medium (DMEM) plus 10% fetal bovine serum on round coverslips coated with poly‐D‐lysine (Sigma, MO, USA) and rat laminin (Invitrogen, Carlsbad, CA, USA) in a 24‐well chamber.

### 2.8 Cell treatment and transfection

Lentiviral‐mediated overexpressing PICK1 vector (pc‐PICK1) or scrambled lentiviral construct (pc‐NC) were designed and produced (GenePharma Co. Ltd, Shanghai, China). Cells in logarithmic phase were seeded into 6‐well plates at a final concentration of 4 × 10^5^/well and cultured for 24 h before transfection. Cell transfection was performed using Lipofectamine 2000 (Invitrogen). Further experiments were carried out at indicated times after transfection. 10 mM FSC231 was used to treat DRG neurons for 30 min, and subsequent experiments were conducted.

### 2.9 RNA extraction and quantitative real‐time polymerase chain reaction

Total RNA was extracted from rat L4‐L6 DRGs or DRG neurons cells using Trizol reagent (Invitrogen) and reverse transcribed into cDNAs using the Reverse Transcription Kit (TaKaRa, Dalian, China). Polymerase chain reaction (PCR) was performed using the SYBR Premix Ex Taq II kit (DRR036A; TaKaRa) and Bio‐Rad CFX 96 real‐time PCR system (Bio‐Rad Laboratories, Hercules, CA, USA). RNA quantities of target genes were calculated using the 2^−∆∆Ct^ method. The final results were normalized to the internal control β‐actin. Primer sequences used in the experiment are shown in Table 1.

**TABLE 1:** Sequences of primers

Gene name	Sequence
PICK1	F: 5′‐TACTAACAGCGAGCTTCCGC‐3′***R: 5′‐GGTTCCGAGAGTTGGAGTGG‐3′
GluA2	F: 5′‐TTTTCGGCAAAATAGGCATAGCAACCTTTTATCA‐3′***R: 5′‐TTTTTGATAAAAGGTTGCTATGCCTATTTTGCCG‐3′
SP	F: 5′‐CAGTCGGATCCTTTCAGGGA‐3′***R: 5′‐CGTTTCCCCATCAGACCGA‐3′
β‐Actin	F: 5′‐AGAGATGGCCACGGCTGCTT‐3′***R: 5′‐ATTTGCGGTGGACGATGGAG‐3′

Abbreviations: F, forward primer; PICK1, protein kinase Cα interacting protein 1; R, reverse primer; SP, substance P.

### 2.10 Western blot

Rat L4‐L6 DRGs or DRG neurons cells were lysed in RIPA buffer (Beyotime) and protein concentration was determined using a BCA kit (Thermo Fisher Scientific). Approximately 30 μg of protein extracts were loaded and separated on sodium dodecyl sulfate‐polyacrylamide (SDS‐PAGE) gels and transferred onto polyvinylidene difluoride (PVDF) membranes (Millipore, Bedford, MA, USA). Nonspecific interactions were blocked with 5% skim milk for 2 h. The membranes were incubated with the following primary antibodies at a 1:1000 dilution overnight at 4°C, followed by the horseradish peroxidase‐conjugated secondary antibodies (Abcam). Proteins were detected with electrochemiluminescence (ECL) reagent (Thermo Fisher Scientific). The following antibodies were used in the study: anti‐PICK1 (ab252543; Abcam), anti‐substance P (S1542; Sigma‐Aldrich), anti‐α‐amino‐3‐hydroxy‐5‐methyl‐4‐isoxazole‐propionic acid receptor 2 (AMPAR; GluA2) (13607; Cell Signaling Technology), anti‐glycogen synthase kinase‐3β (GSK‐3β) (ab93926; Abcam), anti‐phospho‐GSK‐3β (5558; Cell Signaling Technology), extracellular regulated protein kinases1/2 (ERK1/2) (ab184699; Abcam) and anti‐phospho‐ERK1/2 (ab201015; Abcam).

### 2.11 Co‐immunoprecipitation

Total proteins were extracted and clarificated. A portion of the supernatant corresponding to 1 mg of total proteins was precleared for 2 h at 4°C with rabbit antibody immunoglobulin (IgG) (1 μg, ProteinTech Group, Chicago, IL, USA) and protein A+G agarose beads (20 μl, Beyotime Institute of Biotechnology, Jiangsu, China) to get rid of nonspecific binding. Subsequently, the precleared supernatant was incubated with 2 μg of anti‐PICK1 antibody or anti‐GluA2 antibody overnight on a spinning wheel at 4°C, with a parallel containing 2 μg rabbit antibody IgG as negative control, and a certain proportion of supernatant without any antibody (input) was used as a positive control. Afterward, 40 μl of protein A+G agarose beads were dropwise added to bind to the antibodies for 3 h at 4°C. The pellets were spun down at 2500 rpm for 5 min and washed five times with RIPA lysis buffer in sequence. The collected proteins were re‐suspended in 20 μl 1 × sodium dodecyl sulfate‐polyacrylamide gel electrophoresis (SDS‐PAGE) loading buffer and instantaneous centrifuged with high speed to the bottom of the tubes. Following by boiling at 100°C for 5 min, the protein samples separated by SDS‐PAGE and analyzed by Western Blot using the primary antibody of anti‐PICK1 or anti‐GSK‐3β or anti‐GluA2 correspondingly.

### 2.12 Enzyme‐linked immunosorbent assay

For rat DRGs, the tissue homogenate was prepared in homogenizing buffer. The homogenate was centrifuged at 15,000 × *g* for 30 min at 4°C, and the supernatant was collected and stored at −80°C for cytokine measurements. The levels of interleukin (IL)‐6, tumor necrosis factor α (TNF‐α) and IL‐10 were measured with their commercial enzyme‐linked immunosorbent assay (ELISA) kits (R&D Systems, Minneapolis, MN, USA) following the manufacturers’ instructions.

### 2.13 Statistical analysis

The unpaired Student's *t*‐test and one‐way analysis of variance were used to analyze differences among two and multiple groups, respectively. All statistical analyses were performed using SPSS version 20.0 (International Business Machines Corporation, Chicago, USA). *p* < .05 was considered to indicate a statistically significant difference.

## 3 RESULTS

### 3.1 FSC231 alleviates PTL‐induced neuralgia in rats

**FIGURE 1:**
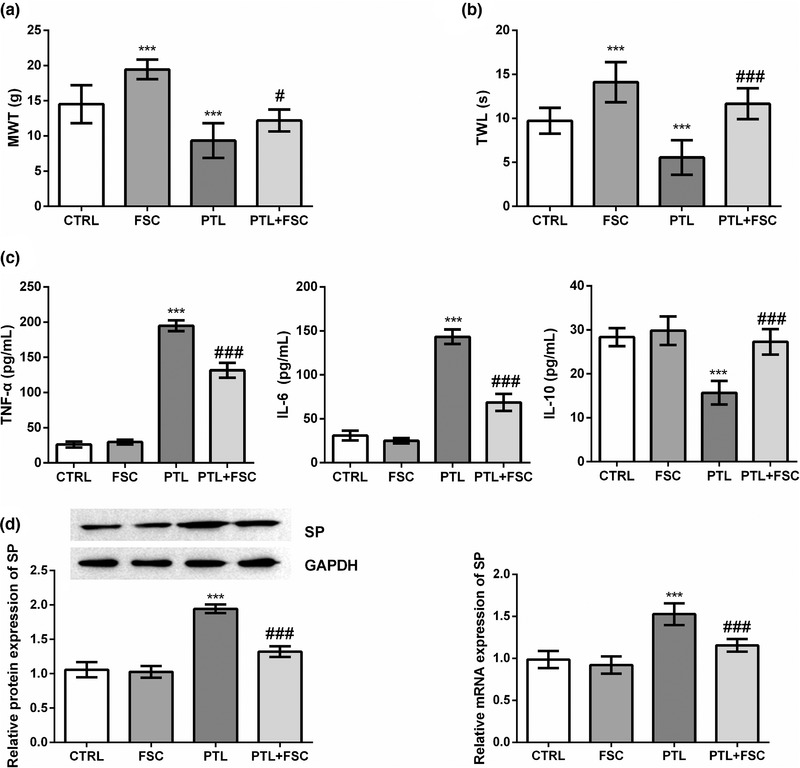
FSC231 alleviates paclitaxel (PTL)‐induced neuralgia in rats. (a) Paw mechanical withdrawal threshold (MWT) was measured before and after modeling for a total of 5 weeks. Compared with the control (CTRL) group, the MWT was significantly increased after FSC231 treatment (*p* < .001) and decreased significantly after PTL treatment (*p* < .001); compared with PTL alone, the combined treatment of FSC231 and PTL alleviated the reduction of MWT in rats induced by PTL (*p* < .05). These data indicated that FSC231 treatment alleviated the decrease in MWT induced by PTL; (b) compared with the CTRL group, FSC231 treatment significantly increased thermal withdrawal latency (TWL) in rats (*p* < .001), PTL treatment significantly decreased TWL (*p* < .001) and FSC231 treatment reversed PTL‐induced TWL shortening (*p* < .001). These data indicated that FSC231 treatment alleviated PTL‐induced TWL shortening; (c) the levels of interleukin‐6 (IL‐6), tumor necrosis factor α (TNF‐α) and IL‐10 were measured with their commercial enzyme‐linked immunosorbent assay (ELISA) kits. Compared with the CTRL group, PTL treatment significantly increased the levels of TNF‐α and IL‐6 (*p* < .001) and significantly decreased the levels of IL‐10 (*p* < .001) in dorsal root ganglion (DRG) of rats, while FSC231 treatment could reverse the changes of inflammatory cytokines caused by PTL (*p* < .001). These data indicated that FSC231 treatment reversed PTL‐induced increases in TNF‐α and IL‐6 levels and decreases in IL‐10 levels; (d) PTL treatment can also significantly increase the levels of substance P (SP) mRNA and protein in DRG (*p* < .001), and FSC231 treatment could also counteract the increase of SP expression level caused by PTL (*p* < .001). These data indicated that the increases in SP mRNA and protein expression levels induced by PTL were offset by FSC231 treatment. ^***^ *p* < .001, compared with the CTRL group; ^#^ *p* < .05, ^###^ *p* < .001, compared with the PTL group

Compared with the CTRL group, the MWT was significantly increased after FSC231 treatment (*p* < .001) and decreased significantly after PTL treatment (*p* < .001) (Figure 1a). Compared with PTL alone, the combined treatment of FSC231 and PTL alleviated the reduction of MWT in rats induced by PTL (*p* < .05) (Figure 1a). Similarly, compared with the CTRL group, FSC231 treatment significantly increased TWL in rats (*p* < .001), PTL treatment significantly decreased TWL (*p* < .001) and FSC231 treatment reversed PTL‐induced TWL shortening (*p* < .001) (Figure 1b). In addition, we extracted the DRG of rats in each group and detected the levels of inflammatory factors and substance P (SP). Compared with the CTRL group, PTL treatment significantly increased the levels of TNF‐α and IL‐6 (*p* < .001) and significantly decreased the levels of IL‐10 (*p* < .001) in DRG of rats, while FSC231 treatment could reverse the changes of inflammatory cytokines caused by PTL (*p* < .001) (Figure 1c). PTL treatment can also significantly increase the levels of SP mRNA and protein in DRG (*p* < .001), and FSC231 treatment could also counteract the increase of SP expression level caused by PTL (*p* < .001) (Figure 1d).

### 3.2 FSC231 activates GSK‐3β and ERK1/2 by inhibiting the interactions of PICK1 with GluA2

**FIGURE 2:**
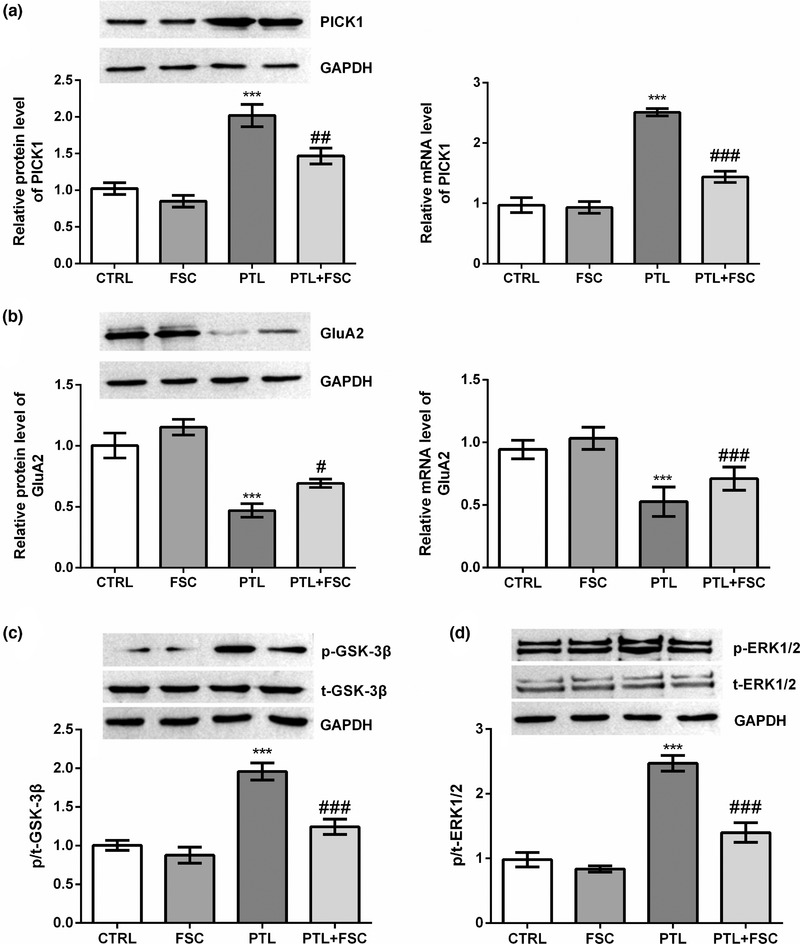
FSC231 reversed paclitaxel (PTL)‐induced increased protein kinase Cα interacting protein 1 (PICK1) levels, decreased GluA2 levels and increased glycogen synthase kinase‐3β (GSK‐3β) and extracellular regulated protein kinases1/2 (ERK1/2) phosphorylation. The mRNA and protein expression levels of related genes were detected by reverse transcription polymerase chain reaction (RT‐PCR) and Western Blot. (a) PICK1 mRNA and protein expression levels were significantly increased in the PTL group compared to the control (CTRL) group (*p* < .001), while FSC231 treatment significantly decreased the expression level of PICK1 (*p* < .001). These data indicated that FSC231 treatment reversed the PTL‐induced PICK1 level increase; (b) compared with the CTRL group, the mRNA and protein expression levels of GluA2 were significantly decreased in the PTL group (*p* < .001), while FSC231 treatment significantly increased the mRNA (*p* < .001) and protein levels of GluA2 (*p* < .05). These data indicated that FSC231 processing reversed PTL‐induced GluA2 reduction; (c and d) compared with the CTRL group, the phosphorylation levels of GSK‐3β and ERK1/2 in dorsal root ganglion (DRG) tissues were significantly increased after PTL treatment (*p* < .001). However, compared with the PTL group, the phosphorylation levels of GSK‐3β and ERK1/2 were partially inhibited after co‐treatment with FSC231 and PTL (*p* < .001). These data indicated that treatment with FSC231 reversed the increase of GSK‐3β and ERK1/2 phosphorylation induced by PTL. ^***^ *p* < .001, compared with the CTRL group; ^#^ *p* < .05, ^##^ *p* < .01, ^###^ *p* < .001, compared with the PTL group

The mRNA and protein expression levels of related genes in DRG were detected by reverse transcription polymerase chain reaction (RT‐PCR) and Western Blot. PICK1 mRNA and protein expression levels were significantly increased in the PTL group compared to the CTRL group (*p* < .001), while FSC231 treatment significantly decreased the expression level of PICK1 (*p* < .001) (Figure 2a). Compared with the CTRL group, the mRNA and protein expression levels of GluA2 were significantly decreased in the PTL group (*p* < .001), while FSC231 treatment significantly increased the mRNA (*p* < .001) and protein levels of GluA2 (*p* < .05) (Figure 2b). Compared with the CTRL group, the phosphorylation levels of GSK‐3β and ERK1/2 in DRG tissues were significantly increased after PTL treatment (*p* < .001) (Figure 2c,d). However, compared with the PTL group, the phosphorylation levels of GSK‐3β and ERK1/2 was partially inhibited after co‐treatment with FSC231 and PTL (*p* < .001) (Figure 2c,d).

**FIGURE 3:**
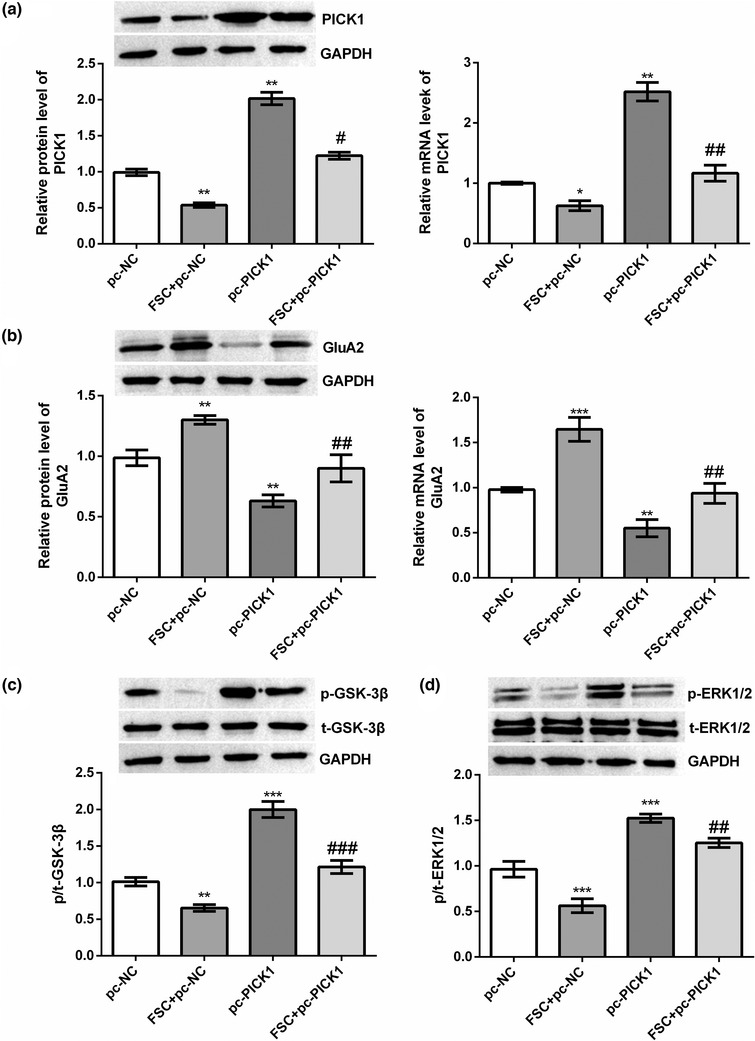
FSC231 could counteract the decreased levels of GluA2 and increased phosphorylation levels of glycogen synthase kinase‐3β (GSK‐3β) and extracellular regulated protein kinases1/2 (ERK1/2) caused by overexpressed protein kinase Cα interacting protein 1 (PICK1). The plasmids were used to overexpress PICK1 in rat dorsal root ganglion (DRG) to investigate the mechanism of action of FSC231. The mRNA and protein expression levels of related genes were detected by reverse transcription polymerase chain reaction (RT‐PCR) and Western Blot. (a) PICK1 was effectively overexpressed by plasmid in DRG, and FSC231 could significantly inhibit PICK1 expression; (b) FSC231 could reverse the decrease in GluA2 expression caused by overexpression PICK1; (c) FSC231 inhibited the increase in GSK‐3β phosphorylation caused by overexpression of PICK1; (D) FSC231 inhibited the increase in ERK1/2 phosphorylation caused by overexpression of PICK1. ^*^ *p* < .05, ^**^ *p* < .01, ^***^ *p* < .001, compared with the pc‐NC group; ^#^ *p* < .05, ^##^ *p* < .01, ^###^ *p* < .001, compared with the pc‐PICK1 group

**FIGURE 4:**
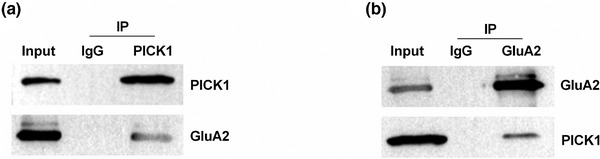
The interaction between protein kinase Cα interacting protein 1 (PICK1) and GluA2 was checked by co‐immunoprecipitation (Co‐IP) experiments. These results demonstrated the interaction between PICK1 and GluA2

We used plasmids to overexpress PICK1 in rat DRG to investigate the mechanism of the action of FSC231. Compared with the CTRL group, pc‐PICK1 plasmid significantly increased the mRNA and protein expression levels of PICK1 in rat DRG (*p* < .01, Figure 3a). FSC231 treatment significantly inhibited PICK1 mRNA and protein expression, both in the CTRL group and in DRG rats with PICK1 overexpression (*p* < .05 or *p* < .01, Figure 3a). Compared with the CTRL group, FSC231 treatment significantly increased the mRNA (*p* < .001) and protein (*p* < .01) expression levels of GluA2, and overexpression PICK1 significantly decreased the expression level of GluA2 (*p* < .01) (Figure 3b). Compared with the pc‐PICK1 group, FSC231 treatment reversed the decrease in GluA2 levels caused by overexpressed PICK1 (*p* < .01, Figure 3b). Compared with the CTRL group, FSC231 treatment significantly reduced the phosphorylation levels of GSK‐3β (*p* < .01) and ERK1/2 (*p* < .001), and overexpression of PICK1 significantly increased the phosphorylation levels of GSK‐3β and ERK1/2 (*p* < .001) (Figure 3c,d). FSC231 treatment significantly inhibited the increased phosphorylation of GSK‐3β (*p* < .001) and ERK1/2 (*p* < .01) caused by overexpression of PICK1 (Figure 3c,d). The results of the co‐immunoprecipitation experiment in Figure 4 demonstrate the interaction between PICK1 and GluA2.

### 3.3 Knocking down PICK1 expression in conjunction with FSC231 treatment significantly alleviated PTL‐induced neuralgia

**FIGURE 5:**
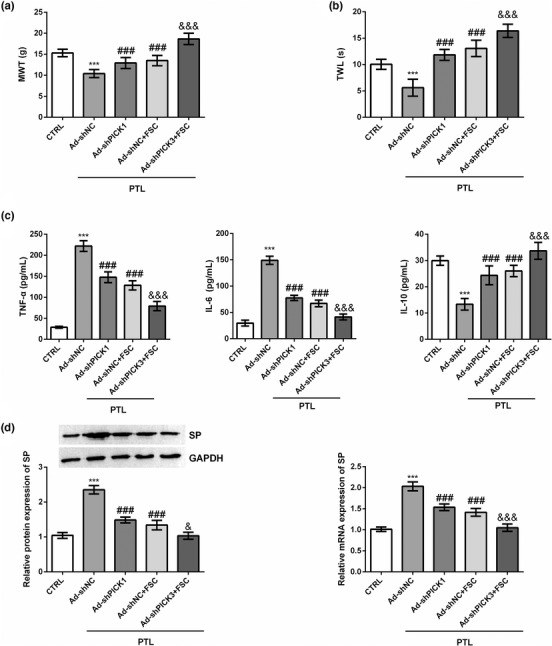
Knocking down protein kinase Cα interacting protein 1 (PICK1) expression in conjunction with FSC231 treatment significantly alleviated paclitaxel (PTL)‐induced neuralgia. The lentiviral vector was used to knock down the expression of PICK1 gene in dorsal root ganglion (DRG). FSC231 treatment was assisted to observe the changes of neuralgia in rats. (a) Silent PICK1 combined with FSC231 therapy significantly alleviated PTL‐induced reduction in mechanical withdrawal threshold (MWT); (b) silent PICK1 combined with FSC231 therapy significantly alleviated PTL‐induced shorting in thermal withdrawal latency (TWL); (c) silent PICK1 combined with FSC231 therapy significantly alleviated PTL‐induced inflammation in DRG; (d) silent PICK1 combined with FSC231 therapy significantly alleviated PTL‐induced release of substance P (SP). ^***^ *p* < .001, compared with the CTRL group; ^###^ *p* < .001, compared with the Ad‐shNC group; ^&^ *p* < .05, ^&&&^ *p* < .001, compared with the Ad‐shNC+FSC group

We used lentiviral vector to knock down the expression of PICK1 gene in DRG, and FSC231 treatment was assisted to observe the changes of neuralgia in rats. MWT and TWL were significantly reduced in rats treated with PTL compared to the CTRL group (*p* < .001, Figure 5a,b). Both FSC231 treatment and silencing PICK1 alleviated PTL‐induced MWT reduction and TWL shortening in rats (*p* < .001, Figure 5a,b). Compared with FSC231 treatment group, silent PICK1 combined with FSC231 treatment had stronger alleviating effect on PTL‐induced MWT reduction and TWL shortening (*p* < .001, Figure 5a,b). PTL treatment also significantly increased the expression levels of TNF‐α and IL‐6 in DRG and significantly inhibited the level of IL‐10 (*p* < .001, Figure 5c). Both FSC231 treatment and silenced PICK1 treatment can alleviate PTL‐induced increases in TNF‐α and IL‐6 levels and decreases in IL‐10 levels in DRG, and the synergistic treatment had a superimposed effect (*p* < .001, Figure 5c). In addition, PTL treatment significantly increased SP mRNA and protein expression levels in DRG (*p* < .001, Figure 5d). Both FSC231 treatment and silent PICK1 could reduce SP expression levels that were elevated due to PTL (*p* < .001, Figure 5d). PICK1 silencing with FSC231 treatment could further significantly reduce SP mRNA (*p* < .001) and protein expression levels in DRG (*p* < .05) (Figure 5d).

### 3.4 FSC231 inhibited the interaction of PICK1 with GluA2 and activated GSK‐3β and ERK1/2 to alleviate PTL‐induced neuralgia

**FIGURE 6:**
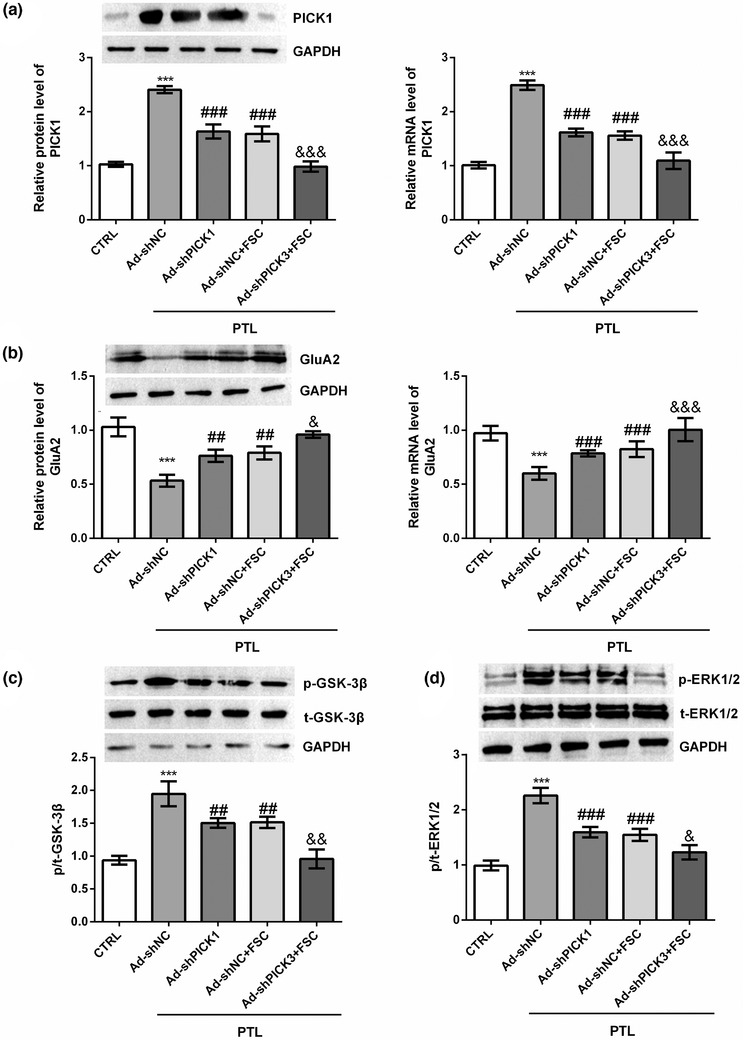
FSC231 inhibited the interaction of protein kinase Cα interacting protein 1 (PICK1) with GluA2 and activated glycogen synthase kinase‐3β (GSK‐3β) and extracellular regulated protein kinases1/2 (ERK1/2). The mRNA and protein expression levels of related genes were detected by reverse transcription polymerase chain reaction (RT‐PCR) and Western Blot. (a) both FSC231 treatment and lentivirus transfection could significantly inhibit paclitaxel (PTL)‐induced PICK1 expression; (b) both FSC231 treatment and lentivirus transfection could reverse the decrease of GluA2 expression induced by PTL and had a cumulative effect; (c) both FSC231 treatment and lentivirus transfection could reduce the increase of GSK‐3β phosphorylation induced by PTL and had a cumulative effect; (d) both FSC231 treatment and lentivirus transfection could reduce the increase of GSK‐3β phosphorylation induced by PTL and had a cumulative effect. ^***^p < .001, compared with the control (CTRL) group; ^##^ *p* < .01, ^###^ *p* < .001, compared with the Ad‐shNC group; ^&^ *p* < .05, ^&&^ *p* < 0.01, ^&&&^ *p* < .001, compared with the Ad‐shNC+FSC group

We detected the mRNA and protein expression levels of related genes in rat DRG. PTL treatment significantly increased the expression level of PICK1 in DRG compared to the CTRL group (*p* < .001, Figure 6a). Both lentivirus treatment and FSC231 treatment could significantly reduce the mRNA and protein expression levels of PICK1, suggesting that FSC231 inhibition of PICK1 and our grouping model were effective (*p* < .001, Figure 6a). FSC231 treatment and lentivirus transfection had a cumulative effect on the inhibition of PICK1 expression level (*p* < .001, Figure 6a). After PTL treatment, the expression level of GluA2 in DRG tissue of mice was significantly decreased (*p* < .001, Figure 6b). Both FSC231 treatment and silent PICK1 could increase the mRNA (*p* < .001) and protein (*p* < .01) expression of GluA2, and co‐treatment with FSC231 and silent PICK1 can further stimulate the mRNA (*p* < .001) and protein (*p* < .05) expression of GluA2 (Figure 6b). PTL increased the phosphorylation levels of GSK‐3β and ERK1/2 in rat DRG tissues compared with the CTRL group (*p* < .001, Figure 6c,d). Both FSC231 and silent PICK1 inhibited GSK‐3β (*p* < .01) and ERK1/2 (*p* < .001) phosphorylation, and the co‐treatment of both further inhibited GSK‐3β (*p* < .01) and ERK1/2 (*p* < .05) phosphorylation (Figure 6c,d).

## 4 DISCUSSION

There are many synapses in nerve cells, and these synapses are the structure of nerve transmission through the transmission of information to achieve the completion of function. Synaptic plasticity is manifested in the increase or decrease of the number of synapses, and this physiological function is the basis of nerve signal transmission. A growing body of evidence suggests that changes in the number of AMPA receptors are closely related to increases or decreases in the number of synapses (Lee et al., 2004). The dynamic transport of AMPA receptors into and out of synapses plays an important role in synaptic transformation and remodeling of learning and memory, but the transport of AMPA receptors cannot be completed independently. PICK1 is one of the proteins that mediate AMPA receptor transport. PICK1 contains a PDZ domain that directly interacts with the GluA2/3 submonomer of the AMPA receptor. The GluA2 submonomer of AMPA receptor mainly blocks the permeability of AMPA receptor Ca^2+^, and the synaptic transmission of GluA2 regulates the permeability of synaptic Ca^2+^, which is dependent on the AMPA receptor (Li et al., 2016). The transfer of GluA2 in the endoplasmic reticulum of the cytoplasm of hippocampal neurons requires the release of internally stored Ca^2+^ and the binding of the calmodulin kinase II to the PDZ protein in PICK1 (Lu et al., 2014). There is evidence that during cerebellar long‐term inhibition, the influx of calcium ions increases, and PICK1 causes a conformational change by binding the PDZ domain to activated protein kinase C (PKC), bringing the PKC closer to GluA2 through its interaction with GluA2 and phosphorylating GluA2 at Ser880 (Hanley, 2008). Phosphorylation of Ser880 of GluA2 by PKC enhances the interaction between PICK1 and GluA2 and facilitates PICK1‐mediated AMPA receptor internalization (Chung et al., 2003).

Our results showed that PTL treatment can significantly reduce MWT, shorten TWL, promote DRG inflammation and release SP, while FSC231 treatment can alleviate the above effects induced by PTL, which is similar to the research of G. Wang et al. (2021). These results confirm that FSC231 has an inhibitory effect on PTL‐induced neuralgia. Overexpression of PICK1 can counteract reduced PICK1 level, increased GluA2 level, and decreased GSK‐3β and ERK1/2 phosphorylation levels caused by FSC231 treatment. The results of Co‐IP confirmed the interactions between PICK1 and GluA2, indicating that FSC231 works by suppressing the interactions of PICK1 with GluA2.

Extracellular signal‐regulated kinase (ERK) 1/2, a member of the mitogen‐activated protein kinase (MAPK) family, is widely expressed in the central nervous system and is also involved in the development, differentiation, proliferation, apoptosis and other physiological and pathological processes of nerve cells (Zou et al., 2019). ERK1/2 is closely related to the function of the central nervous system (Carriba & Davies, 2021; Ibrahim et al., 2021). It can affect the growth and development of nerve cells axon, excitability of nerve cells and plasticity of nerve cells between synapses through intracellular signal transmission, gene expression and protein synthesis (Medina & Viola, 2018; Singh et al., 2020). Some studies show that the occurrence and development of PTL‐induced neuralgia is also closely related to ERK activation (Janes, Little, et al., 2014). After nerve and spinal cord injury, increased ERK phosphorylation in spinal microglial cells and astrocytes, and activated ERK are essential for inflammation caused by intracellular signal transduction of glial cells and the production of early pain mediators, suggesting that ERK plays an important role in the generation and maintenance of neuralgia caused by nerve injury (Ji et al., 2009). In addition, enhanced nuclear factor kappa‐B (NF‐κB) signaling pathway was found in the lumbar spinal cord in PTL‐induced neuropathic pain model, promoting nuclear translocation of NF‐κB p65 and increased phosphorylation of ERK1/2 (Janes, Esposito, et al., 2014).

Glycogen synthase kinase‐3β (GSK‐3β) is highly expressed in the central nervous system and is widely involved in physiological processes such as cell development, differentiation, proliferation and apoptosis (Ibrahim et al., 2021; Singh et al., 2020). It is the hub of multiple intracellular signaling pathways and transduction of intracellular signals (Beurel et al., 2015). GSK‐3β has two special phosphorylation sites through which the phosphorylation of GSK‐3β protein can change the activity of GSK‐3β protein, and then participate in various physiological and pathological processes of nerve cells. GSK‐3β normally remains active, but GSK‐3β can be inactivated by phosphorylation of MAPK signaling pathway (Ko & Lee, 2016). Recently, it has been suggested that under various stimuli, GSK‐3β can play a regulatory role as downstream regulatory proteins of different signaling pathways, and when the expression of GSK‐3β protein is dysregulated, it will promote the occurrence of diabetic nervous system diseases and tumors and other diseases (Perales‐Linares & Navas‐Martin, 2013). Studies have found that Ser416 is a very important phosphorylation site in PICK1 and plays a key role in regulating the interaction between PICK1 and GluA2. GSK‐3β mediates PICK1 phosphorylation, which requires binding of GluA2 (Yagishita et al., 2015). Pharmacologic inhibition of GSK‐3β phosphorylation has also been shown to decrease the phosphorylation of PICK1 and increase the expression of GluA2, thereby increasing the neuro‐enhancing effect of alcohol on mice (Faccidomo et al., 2020). It has been reported that the phosphorylation level of ERK1/2 is regulated by proteins containing the PDZ domain (Chung et al., 2003). Of interest, phosphorylation of ERK1/2 phosphorylates more than 150 downstream protein molecules, including multiple nuclear transcription factors and GSK‐3β (Luo et al., 2016). To sum up, the interaction mechanisms among PICK1, GluA2, GSK‐3β and ERK1/2 are intricate and cannot be fully elucidated until now.

Our study confirmed that overexpression of PICK1 can reduce the expression of GluA2 and increase the phosphorylation levels of GSK‐3β and ERK1/2, and that PICK1 and GluA2 interact. At the same time, FSC231 treatment or silencing PICK1 has the opposite effect. In conclusion, FSC231 alleviated PTL‐induced neuralgia by inhibiting the interactions between PICK1 and GluA2 and activated GSK‐3β and ERK1/2. This study provides new experimental data on the mechanism of FSC231's analgesic action in PTL‐induced neuralgia by inhibiting PICK1.

PTL‐induced neuralgia is dose‐dependent. In future studies, we can further study the effect and mechanism of FSC231 on different doses of PTL‐induced neuralgia. Further, we need to determine the dosage of FSC231 when PTL causes varying degrees of neuralgia.

## FUNDING INFORMATION

The Research Foundation of Health Commission of Hubei Province, China (No. WJ2021Q009), Innovative Research Program for Graduates of Hubei University of Medicine (No. YC2021042), and Science and Technology Project of Shiyan City (No. 21Y69).

### PEER REVIEW

The peer review history for this article is available at https://publons.com/publon/10.1002/brb3.2380.

## Data Availability

The datasets generated and analyzed during the current study are available from the corresponding author on reasonable request.
